# Pathways into child and adolescent mental health services for anxiety and depression: The role of social and educational factors

**DOI:** 10.1007/s00787-026-03084-6

**Published:** 2026-06-13

**Authors:** Yeosun Yoon, Jessica Deighton, Alice Wickersham, Zoe Chui, Stephanie Lewis, Julian Edbrooke-Childs, David Osborn, Essi Viding, Amelia Jewell, Johnny Downs

**Affiliations:** 1 University of Cambridge, Cambridge, UK; 2 Anna Freud Centre, Evidence Based Practice Unit, London, UK; 3 University College London, London, UK; 4 King’s College London, London, UK; 5 South London and Maudsley NHS Foundation Trust, London, UK

**Keywords:** Adolescence, Mental health, Referral pathways, Education

## Abstract

Anxiety and depression are increasingly prevalent among young people and significantly impact their lives. However, research on the referral pathways into Child and Adolescent Mental Health Services (CAMHS) for young people with these disorders, and how these pathways vary according to socio-demographic characteristics, remains limited. Moreover, few studies have examined whether educational factors are associated with these referral pathways. Therefore, this study aimed to examine the relationships of sociodemographic and educational characteristics with CAMHS referral pathways for anxiety disorders and depression. We used routinely collected educational data linked with CAMHS records from South London and Maudsley NHS Foundation Trust, focusing on young people aged 12–17 years with clinical diagnoses of anxiety disorders and depression (*N* = 4,169). Using multinominal logistic regression, we identified that referral pathways to CAMHS are associated with various sociodemographic and educational factors. Specifically, youth with lower educational attainment, school exclusion, or SEN status, were more likely to be referred to CAMHS through education. This suggests that educational services may be a key referral pathway for young people in need of mental health support for anxiety and/or depression and referring to CAMHS. However, Black young people, those who speak English as an additional language (EAL), individuals eligible for free school meals (FSM), and young people under the care of local authorities were more likely to be referred via social care or youth justice services. Also, older young people, females and those under the care of local authority showed a higher likelihood of being referred via emergency services rather than primary care. These findings highlight that efforts to identify problems earlier through primary care or education services could benefit for those young people who are more frequently referred via emergency or social care/youth justice routes. Further research is needed to examine the potential under-utilisation of certain referral pathways by specific groups, in order to improve accessibility and ensure that more young people can access effective mental health care.

## Introduction

There is increasing prevalence and a growing awareness of mental health difficulties in children and young people, particularly anxiety and depression [1]. For example, recent research estimates that around 18% of children and young people in England meet clinical thresholds for anxiety and depression [2]. These difficulties can lead to significant distress, functional impairment, and self-harm [3]. Thus, timely access to appropriate mental health support for children and young people is critical.

In the UK, evidence-based treatments for mental health disorders in children and young people are most commonly accessed through referral to Child and Adolescent Mental Health Services (CAMHS). Access to these services varies; children and young people may be referred via primary care (e.g., general practitioners) or through educational settings, such as schools. Other referral pathways include emergency services, social care, and youth justice services, reflecting distinct referral pathways. For example, emergency services referrals often occur in crisis situation [4], social care referral typically involves children receiving statutory or targeted support from children’s service due to safeguarding concerns, family difficulties or risks to wellbeing [5]. Youth justice referral may arise when children involved with the justice system are identified as having unmet mental health needs and are referred for support [6].

Initial evidence has suggested that referral routes into CAMHS vary according to sociodemographic characteristics. For example, Edbrooke-Childs and colleagues found that referral pathways vary by age, gender and ethnicity [7]. In particular, children from black and ethnic minority backgrounds were more likely to be referred through education, social services, and youth justice than primary care, compared to White children [8]. Similarly, Chui and colleagues identified ethnic differences in access to mental health services among adolescents and young adults aged 12–29 years, with Black young people were more likely than their White peers to be referred via secondary health, social care, or criminal justice services rather than through primary care.

However, important gaps remain in the evidence base. Few studies have examined how referral pathways are associated with a wider range of social and educational factors, such as socio-economic status, or educational experiences, despite their likely influence on access to care. Previous research has predominantly focused on overall CAMHS referrals or service access [8–11], highlighting substantial unmet needs, but capturing heterogenous population with a broad range of mental health conditions.

Referral routes into CAMHS may also vary by presenting mental health problem, highlighting the importance of condition-specific analysis. However, there remains limited research on factors associated with referral routes for children and young people with specific conditions. The NHS England ADHD task force has recently examined inequalities in access/assessment and referral pathways for attention deficit hyperactivity disorders across services [12]. Nevertheless, evidence remains limited for other common conditions, such as anxiety disorders and depression. This study therefore aimed to examine associations between socioeconomic and educational factors and routes of referral into CAMHS among children and young people with anxiety or depression.

## Method

### Study design and data source

This retrospective cohort study utilised an existing data linkage between two administrative health and education datasets. We sourced mental healthcare data from South London and Maudsley NHS Foundation Trust (SLaM), one of Europe’s largest mental healthcare providers [13]. SLaM is the sole provider of state-funded specialist mental healthcare for the population of four South London boroughs (Croydon, Lambeth, Lewisham, Southwark; total population around1.3 million). Some patients are also referred to SLaM from outside the catchment area to National and Specialist teams [14]. We obtained de-identified electronic health records from SLaM, via the Clinical Record Interactive Search system (CRIS).

We sourced education data from the UK’s National Pupil Database (NPD), which contains routinely collected education and social care data for all pupils in England’s state-maintained schools. CRIS records for a cohort of young people referred to SLaM CAMHS between 2007 and 2013 have been linked with the NPD. This linkage is described in detail elsewhere [15, 16].

### Sample

We included young people aged 12–17 years at the time of their CAMHS first referral, who were accepted for assessment by SLaM CAMHS between 1st January 2008 and 31st December 2013, and were subsequently diagnosed with an anxiety or non-psychotic depressive disorder (ICD-10 F32-F34, F38-43, F48, F91-93). We excluded young people who were also diagnosed with bipolar affective disorder, mania, schizophrenia spectrum or psychotic depressive disorders (ICD-10 F20-F32) due to the difference in care pathways and services for these conditions. In total, *n* = 4,813 young people with linked health and education data were eligible for inclusion in this study, of whom *n* = 4,169 (86.6%) had a complete set of variables without any missing information for our analysis.

### Referral source

We considered seven categories of CAMHS referral source as the outcome variable in our analysis: primary care, education, child health (i.e., paediatrics), emergency department, other mental health services (including other regions’ state-funded services, independent/private sector, voluntary sector), social care/youth justice (including social services, courts, police, probation, and youth justice services), and other referral routes (including self-referral, medium security/forensic services, other clinical agencies, and others) (see Table 1). Some sources were combined in these categories due to small cell sizes including social care and youth justice referrals.

### Sociodemographic characteristics

Age at first referral, gender and ethnicity were extracted from CRIS, with missing values supplemented from NPD school census records. Ethnic groups were categorised as Asian, Black, Mixed, White, Other, and Unknown. English as an additional language, free school meals eligibility, and neighbourhood deprivation, all in the academic year prior to first CAMHS referral, were obtained from NPD school census records. Free school meals eligibility reflects children in receipt of, or whose parents/carers are in receipt of, financial and other income supports, and is therefore used as a proxy measure for socioeconomic status [17]. Levels of neighbourhood deprivation were based on the young person’s home neighbourhood Index of Multiple Deprivation national quintile, a composite measure of multiple domains of deprivation across England. Looked After Child (LAC) status at any time prior to the first referral was also derived from NPD records. In the UK, LAC refers to children under the care of local authority, including those in foster care, residential care or other state-supported arrangements [18].

### Educational factors

Special Education Needs (SEN) status and low school attendance were derived from NPD records relating to the academic year prior to first CAMHS referral. SEN status is assigned to young people who have a learning difficulty or disability that requires them to receive additional support beyond that typically provided to pupils [19]. Low school attendance was defined as missing 15% of sessions or more, based on the Department of Education’s definition up until September 2015 [20]. Records of having any school exclusions (including any fixed-period or permanent exclusions) were extracted from NPD data for the academic year that immediately preceded the first referral during the study period. Educational attainment data were extracted from the NPD in the form of Key Stage 2 standardised attainment test (SATs), typically assessed at the end of Year 6 when pupils are aged 10–11 years. The average total test scores across English, Mathematics and Science were used to calculate ranked z-scores which were then grouped into quintiles using the entire pupil population. Pupils working below the required test levels, and therefore not receiving test marks, were allocated to the lowest quintile.

### Statistical analysis

We used multinominal logistic regression to estimate the associations between sociodemographic characteristics and educational factors (exposures) with referral routes into CAMHS (outcome). Results are presented as relative risk ratios (RRR) with 95% confidence intervals (CIs) [21]. We fitted a fully adjusted model including age, gender, sociodemographic characteristics (ethnicity, English as an additional language, free school meal eligibility, neighbourhood deprivation and LAC status), and education factors (SEN status, low school attendance, school exclusion, and educational attainment). For the outcome variable, the primary care referral route was selected as the reference category, as it represented the largest group. Initially, multilevel multinominal logistic regression modelling was attempted to account for the possible effect of clustering at the young person’s home local authority level. However, very little of the variance in referral routes was explained by local authority (interclass correlation coefficient < 2%), so single-level multinominal logistic regression was applied. All analyses were conducted in Stata 15 [22].

## Results

### Sample characteristics

Sample characteristics are shown in Table 1. In summary, the sample of 4,169 adolescents with anxiety or depressive disorder included in our study had a mean age of 14.2 years, and was mostly female (65.7%), of White ethnicity (59.8%), and spoke English as a first language (86.5%). Just under a third (27.7%) were eligible for free school meals, and over two-thirds (68.0%) resided in neighbourhoods in the top two quintiles with highest deprivation levels nationally. Around 5% of the sample had been looked after children, under local authority care, before their first referral. 38.8% of the sample had Special Educational Needs status, 24.1% had low school attendance, and 21.7% had experienced at least one school exclusion in the academic year prior to their first referral. Nearly half (48.3%) were within the lowest two quintiles for educational attainment in key stage 2 (see Table 2 for comparison with national population estimates). The most common referral source was primary care (40.7%), followed by other referral source (25.5%), and education (10.2%). The number of referrals per year ranged from 524 in 2008 to 773 in 2013. The majority (70.7%) of referred young people were from the four South London boroughs.

**Table 1:** Sample characteristics

Characteristics	% (*n*)
Age at referral, years, mean (SD)	14.2 (1.5)
Gender	
Male	34.3 (1,431)
Female	65.7 (2,738)
Ethnicity	
Asian	4.1 (169)
Black	19.0 (792)
Mixed	12.9 (536)
White	59.8 (2,495)
Other ethnic group	2.3 (98)
Unknown	1.9 (79)
English as additional language	
Yes	13.5 (563)
No	86.5 (3,606)
Free school meal eligibility in the academic year prior to referral	
Yes	27.7 (1,156)
No	72.3 (3,013)
Neighbourhood deprivation, national quintile	
Most deprived	32.2 (1,342)
2	35.8 (1,492)
3	16.0 (666)
4	9.6 (400)
Least deprived	6.4 (269)
Looked After Child (LAC) status, ever prior to referral	
Yes	5.1 (213)
No	94.9 (3,956)
Special Educational Needs (SEN) status in the academic year prior to referral	
Yes	38.8 (1,616)
No	61.2 (2,553)
Low school attendance in the academic year prior to referral	
Yes	24.1 (1,005)
No	75.9 (3,164)
School exclusion in the academic year prior to referral	
Yes	21.7 (906)
No	78.3 (3,263)
Educational attainment in Key Stage 2, quintile	
Lowest	28.3 (1,178)
2	20.0 (835)
3	18.8 (784)
4	18.6 (776)
Highest	14.3 (596)
CAMHS referral source	
Primary care	40.7 (1,698)
Education	10.2 (427)
Child health	6.5 (272)
Hospital emergency department	8.9 (370)
Other mental health services	4.2 (174)
Social care/youth justice	3.9 (164)
Other referral source	25.5 (1,064)
Referral year	
2008	12.6 (524)
2009	16.8 (700)
2010	18.0 (749)
2011	16.9 (703)
2012	17.3 (720)
2013	18.5 (773)
Home local authority	
Croydon	22.2 (926)
Lambeth	15.3 (637)
Lewisham	28.9 (1,206)
Southwark	14.3(597)
Other	29.3 (803)

*N* = 4196

**Table 2:** CAMHS sample compared with national statistics

Characteristics	CAMHS sample (%)	England (%)
Free school Meal eligibility	27.7	16.3
Looked after children	5.1	0.6
SEN	38.8	19.0
Low school attendance	24.1	6.5
School exclusion	21.7	5.3

National reference estimates were drawn from contemporaneous Department for Education 2012/13 statistics [23﻿, 24] and are provided as contextual reference points

### Factors associated with CAMHS referral source

Associations between sociodemographic characteristics, educational factors, and referral routes into CAMHS are summarised in Table 3. Findings are narratively summarised for each referral route below, as compared to referral from primary care.

#### Education

Compared to referral from primary care, younger children, those from Asian or Black ethnic groups, children with SEN status, those who experienced school exclusions, or children with lower key stage 2 attainment had higher relative risk of being referred via education services.

#### Child health

Compared with referral from primary care, males, children living in the least deprived neighbourhood quintile, those who had been in local authority care, children with SEN status, or those with lower Key Stage 2 attainment had higher relatively risk of being referred via child health services.

#### Hospital emergency services

Compared with referral from primary care, older children, females, children from Asian or Black ethnic groups, those who had been in local authority care, or children who experienced school exclusion had higher relative risk of being referred via hospital emergency services.

#### Other mental health services

Compared with referral from primary care, children from Black ethnic groups had lower relative risk of being referred via other mental health services compared with White children, while those living in less deprived neighbourhood quintiles, children with SEN status, low school attendance, or school exclusion had higher relative risk of being referred via other mental health services.

#### Social care/youth justice services

Compared with referral from primary care, children from Black ethnic groups, those spoke English as an additional language, children eligible for free school meals, those who had been in local authority care, children with lower school attendance or who experienced school exclusion had higher relative risk of being referred via social care/youth justice services.

#### Other referral routes

Compared with referral from primary care, Black ethnic groups, older children, and children with indicators of educational disadvantage (e.g., SEN status, lower attainment, low attendance or a history of school exclusion) had a higher relative risk of being referred via other routes. Children from Mixed ethnic groups showed a lower relative risk.

**Table 3:** Associations of socio-demographic characteristics and educational factors with CAMHS referral source

	A. Education vs. Primary care				B. Child health vs. Primary care				C. Hospital emergency vs. Primary care				D. Other mental health vs. Primary care				E. Social care/Youth justice vs. Primary care				F. Other vs. Primary care			
	RRR (95% CI)				RRR (95% CI)				RRR (95% CI)				RRR (95% CI)				RRR (95% CI)				RRR (95% CI)			
Sociodemographic characteristics																								
Age at referral, years	**0.76** (0.71–0.83)***				0.95 (0.87–1.04)				**1.34** (1.24–1.46)***				1.01 (0.90–1.13)				0.95 (0.84–1.07)				**1.10** (1.05–1.17)***			
Female gender	1.18 (0.93–1.50)				**0.75** (0.57–0.98)*				**2.44** (1.82–3.28)***				1.19 (0.84–1.69)				0.91 (0.64–1.31)				1.02 (0.86–1.21)			
Ethnicity (ref. White)																								
Asian	**1.85** (1.07–3.21)*				1.46 (0.76–2.80)				**2.12** (1.23–3.66)*				1.21 (0.50–2.93)				1.68 (0.69–4.10)				0.93 (0.58–1.50)			
Black	**1.49** (1.12–1.98)*				1.00 (0.68–1.47)				**2.01** (1.49–2.71)***				**0.26** (0.11–0.60)*				**2.45** (1.61–3.73)***				**1.34** (1.07–1.68)*			
Mixed	1.18 (0.86–1.62)				0.82 (0.54–1.26)				1.16 (0.82–1.66)				0.56 (0.31–1.02)				1.39 (0.84–2.30)				**0.74** (0.57–0.96)*			
Unknown	0.67 (0.28–1.63)				0.33 (0.08–1.41)				1.24 (0.56–2.75)				0.20 (0.03–1.48)				1.32 (0.38–4.56)				0.67 (0.37–1.20)			
Other ethnic group	0.35 (0.12–1.00)				1.30 (0.60–2.81)				1.37 (0.68–2.78)				0.26 (0.03–1.99)				0.78 (0.22–2.76)				0.97 (0.55–1.69)			
English as additional language	1.16 (0.82–1.63)				1.44 (0.96–2.16)				1.29 (0.91–1.81)				1.00 (0.51–1.98)				**1.77** (1.09–2.87)*				0.93 (0.71–1.22)			
Free school meals eligibility	1.23 (0.96–1.57)				1.28 (0.94–1.72)				1.11 (0.85–1.46)				0.82 (0.53–1.28)				**1.67** (1.17–2.39)*				1.18 (0.97–1.43)			
Neighbourhood deprivation national quintile (ref. most deprived)																								
2^nd^	0.93 (0.73–1.20)				0.82 (0.59–1.13)				0.85 (0.65–1.11)				1.04 (0.61–1.77)				1.39 (0.95–2.04)				0.87 (0.71–1.06)			
3^rd^	1.26 (0.91–1.75)				1.16 (0.77–1.76)				0.97 (0.68–1.39)				**3.00** (1.74–5.18)***				0.91 (0.50–1.64)				**1.40** (1.10–1.79)**			
4^th^	0.74 (0.44–1.23)				1.44 (0.87–2.37)				**0.43** (0.23–0.80)*				**6.31** (3.65–10.9)***				0.49 (0.17–1.42)				**2.11** (1.58–2.80)***			
5^th^ (least deprived)	0.97 (0.50–1.88)				**3.80** (2.25–6.41)***				1.18 (0.64–2.18)				**11.74**(6.41–21.5)***				1.31 (0.47–3.61)				**3.18** (2.23–4.55)***			
Looked After Child (LAC) status	1.08 (0.57–2.06)				**2.17** (1.17–4.04)*				**1.90** (1.01–3.59)*				1.80 (0.85–3.79)				**9.54** (5.65–16.1)***				**2.91** (1.91–4.44)***			
Educational factors																								
Special Education Needs (SEN) status	**1.29** (1.01–1.66)***				**1.96** (1.45–2.66)***				0.99 (0.76–1.31)				**2.4** (1.64–3.53)***				1.29 (0.88–1.89)				**1.36** (1.13–1.64)**			
Low school attendance	0.94 (0.71–1.25)				1.16 (0.85–1.60)				1.05 (0.78–1.40)				**1.81** (1.26–2.59)**				**1.82** (1.24–2.66)*				**1.53** (1.26–1.85)***			
School exclusion	**1.87** (1.42–2.45)*				1.11 (0.78–1.56)				**2.01** (1.50–2.70)***				**2.44** (1.64–3.62)***				**3.70** (2.55–5.37)***				**1.50** (1.22–1.86)***			
Educational attainment (Key Stage 2, ref. highest quintile: 5th )																								
1^st^ (Lowest quintile)	**2.02** (1.32–3.10)**				**2.66** (2.62**–**4.38)***				1.37 (0.90–2.08)				1.47 (0.86–2.53)				2.1 (0.97–4.53)				**1.67** (1.26–2.23)***			
2nd	**1.70** (1.12–2.59)*				1.09 (0.63–1.87)				1.16 (0.77–1.75)				0.73 (0.41–1.32)				2.05 (0.95–4.4)				**1.34** (1.02–1.78)*			
3rd	**1.70** (1.12–2.58)*				1.37 (0.81–2.34)				1.41 (0.95–2.10)				0.95 (0.54–1.68)				1.38 (0.62–3.08)				**1.35** (1.02–1.79)*			
4th	1.41 (0.92–2.15)				1.39 (0.83–2.33)				1.22 (0.82–1.82)				0.62 (0.34–1.13)				1.49 (0.66–3.35)				1.10 (0.83–1.45)			

*N =* 4169, ***p < 0.001, **p < 0.01, * *p* < 0.05

## Discussion

By capitalising on a large data linkage between health and education datasets for a diverse population in South London, this study examined CAMHS referral sources for young people with diagnoses of anxiety disorders or depression. The findings highlight the important role that sociodemographic characteristics and educational factors play in referral pathways to CAMHS for these young people. To our knowledge, this is the first study examining the association between a broad range of sociodemographic characteristics, educational factors and CAMHS referral source.

Our findings demonstrate that academically vulnerable youth, such as those with lower educational attainment and those with SEN status, were more likely to be referred to CAMHS through education and child health services rather than primary care. As these young people often receive educational support, and have neurodevelopmental conditions requiring child health services [25], this implies the education and child health systems are positively identifying when pupils experience difficulties academically or behaviourally and able to refer them for mental health support. This suggests that educational systems play an important role in recognising and addressing mental health problems appropriately, particularly for those who might otherwise face difficulties in accessing mental health services through primary care routes.

In addition, young people with a lower school attendance or exclusion records were more likely to be referred to CAMHS through other mental health services or social care/youth justice services rather than primary care services. This might be related with symptoms associated with anxiety and depression contributing to school refusal, as well as disruptive behaviour leading to exclusion [26]. Such difficulties could be more likely to be recognised by other mental health services or social care/youth justice services than by primary care services.

In terms of other sociodemographic factors, our findings align with previous research indicating that younger individuals are more likely to be referred to CAMHS through the education system rather than primary care [7, 8]. This may reflect the crucial role of school-based support systems in early identification of and response to the mental health needs of younger adolescents. In contrast, older adolescent, who may have left school or prefer to manage problems independently, are more likely to be referred via hospital emergency services. This may be consistent with their developmental shifts towards greater autonomy and reduced self-seeking [27], which has been recognised as one of the barriers of early identification and intervention [28]. Referral hesitancy near the upper age boundary of CAMHS may also contribute to these patterns [29].

Furthermore, we found Asian and Black young people were more likely to be referred to CAMHS through education systems and hospital emergency services compared to their White peers. This may suggest that ethnic minority groups might be less likely to utilise primary care services for mental health difficulties, or that primary care clinicians may be less likely to recognise the need for referrals within these groups [8, 9, 30]. Previous research conducted in southeast London has reported variations in help-seeking behaviours across ethnic groups, with Asian and Black African individuals less likely to seek formal support through primary care [31]. Additionally, evidence indicates that primary care services may be less effective in detecting mental health problems in Black Caribbean individuals [32].

Compared with their peers, Black young people, those who speak English as an additional language, those eligible for free school meals, and those in the care of local authorities were more likely to be referred to CAMHS via social care or youth justice services rather than primary care. Referrals from these pathways are often associated with crisis presentations and, in some cases, involuntary admission, reflecting greater severity at presentation [7, 9, 30]. A recent rapid review [33], alongside quantitative evidence from South East London residents [34], suggests that experiences of systemic racial discrimination and lack of trust in mental health services may reduce engagement of voluntary mental health care among ethnic minority groups. Similarly, Andersen and colleagues report that language barrier may impede access to CAMHS [35]. In addition, qualitative evidence from parents and carer of children in care indicated that perceived lack of professional recognition of parental challenges, and feeling of blame regarding children’s mental health difficulties, may act as barrier to seeking support [36]. Therefore, further research would be beneficial to examine the mechanisms underlying disparities in referral pathways, including structural, linguistic, and relational barriers influencing pathways into CAMHS, particularly among ethnic minority young people and those involved with social care services. Improved understanding of these mechanisms may inform development of more equitable and accessible mental health services.

Finally, neighbourhood deprivation also appeared to play an important role in shaping referral pathways to CAMHS among young people with anxiety or depression. Compared to those living in deprived areas, young people residing in relatively less deprived neighbourhoods were more likely to be referred to CAMHS through child health, and other mental health services rather than primary care services. This patterns is consistent with recent evidence that children and young people living in more deprived contexts experience greater need but poorer access to CAMHS [11]. This reflects the inverse intervention law, whereby those with the greater need for service often less likely to receive appropriate or timely intervention [37].

### Strengths and limitations

The use of a whole-population data linkage between mental health and education records is a key strength of this study. It allowed us to capture a wide range of sociodemographic and educational characteristics when examining the referral pathways to CAMHS for children and young people with anxiety and/or depression.

However, several limitations should be considered when interpreting these findings. This study draws on data between 2008 and 2013, and therefore primarily reflects pre-pandemic patterns of accessing CAMHS. Given potential changes in mental health needs, help-seeking behaviours, and service delivery since the COVID-19 pandemic, referral pathways may have shifted to some extent. Nonetheless, documenting pre-pandemic referral patterns provides an important baseline for future research to explore evolving demand and service provision.

Furthermore, as the study sample was drawn from youth mental health services from South London, the representativeness of the findings for CAMHS beyond this area is unknown. Future research in other settings or regions should be conducted to determine whether these findings are replicated. Also, the ‘other’ referral route grouping includes a heterogeneous group of referral pathways, which complicates the interpretation of findings for this group.

### Conclusions

This study underscores the significant role that socioeconomic and educational factors play in CAMHS referral routes for young people with anxiety disorders or depression. Our findings highlight the need for tailored referral pathways that take into account both educational vulnerabilities and sociodemographic characteristics. The educational services appear to be positively identifying young people who need mental health support with anxiety and/or depression and referring them for further services. However, early identification of mental health problems through primary care or education services could be more beneficial for those young people who have been referred via emergency or social care/youth justice routes. This suggests that greater attention should be given to ensuring that young people from disadvantaged or vulnerable backgrounds can access mental health support in a timely and effective manner, consistent with recent recommendations to improve CAMHS referral pathways and access to care [38, 39]. The growing number of home-schooled children and young people may also present additional challenges for identification through school-based referral pathways.

## Data Availability

This study used administrative data. We analysed de‑identified data from South London and Maudsley NHS Foundation Trust (SLaM) CAMHS records linked with the Department for Education’s National Pupil Database (NPD). The detail information to access this linked data is from here: https://www.maudsleybrc.nihr.ac.uk/facilities/clinical-record-interactive-search-cris/cris-data-linkages/.
